# Supplementary material for: Complex‐centric proteome profiling by SEC‐SWATH‐MS
Source: Mol Syst Biol. 2019 Jan 14;15(1):e8438. doi: 10.15252/msb.20188438 (PMC6346213; doi:10.15252/msb.20188438)
Supplement: Supplementary file 8 — Dataset EV7 [file MSB-15-e8438-s008.zip › feature_plots_string/P09104.pdf]

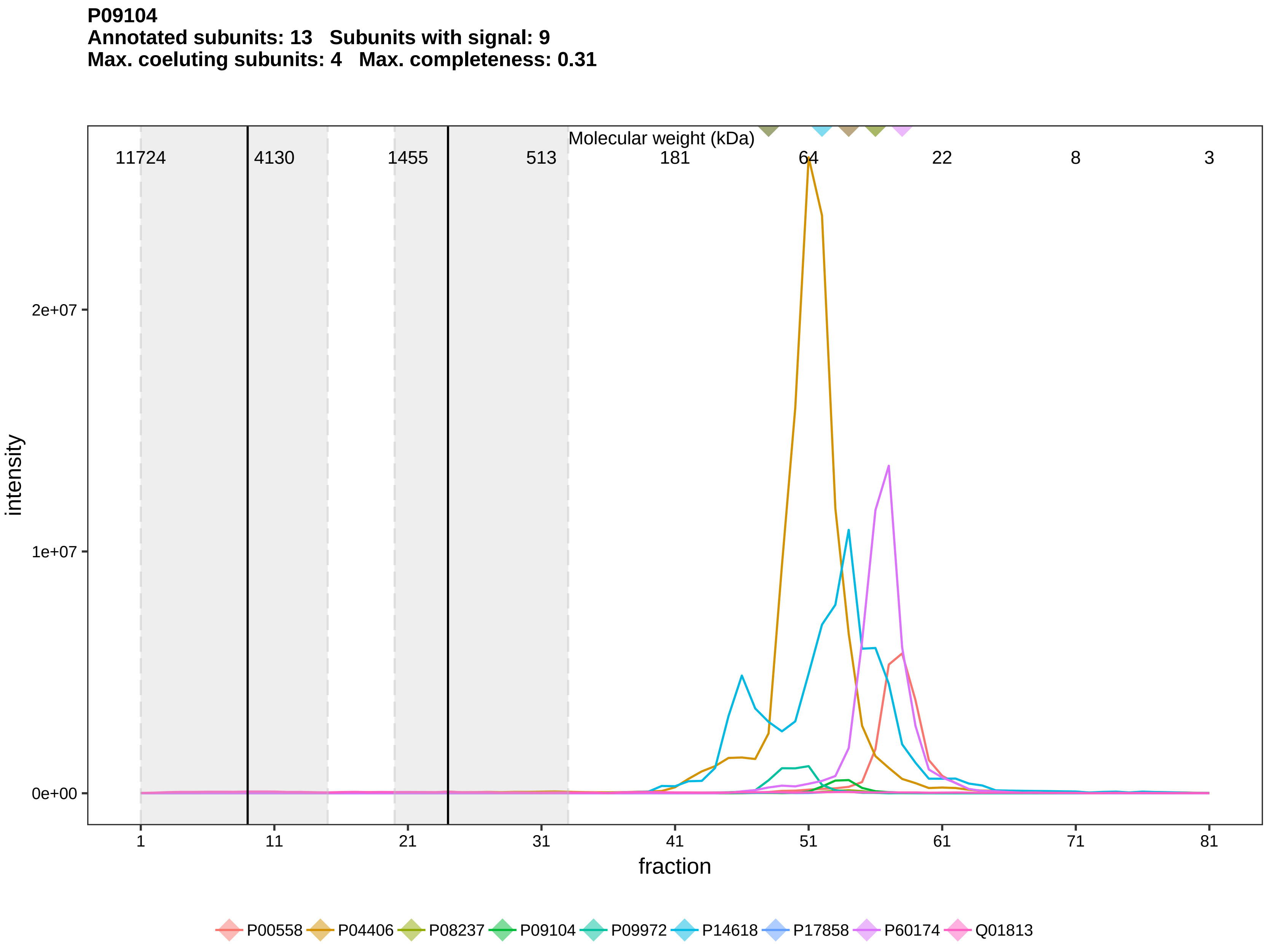
P09104
Annotated subunits: 13 Subunits with signal: 9
Max. coeluting subunits: 4 Max. completeness: 0.31
Molecular weight (kDa)
11724
4130
1455
513
181
64
22
8
3
2e+07
1e+07
0e+00
intensity
1
11
21
31
41
51
61
71
81
fraction
P00558
P04406
P08237
P09104
P09972
P14618
P17858
P60174
Q01813
